# Fatty Acids and Starch Identification within Minute Archaeological Fragments: Qualitative Investigation for Assessing Feasibility

**DOI:** 10.3390/foods13071090

**Published:** 2024-04-02

**Authors:** Roberto Ordoñez-Araque, Luis Ramos-Guerrero, Paul Vargas-Jentzsch, Martha Romero-Bastidas, Nicolás Rodríguez-Herrera, Rubén Vallejo-Holguín, Camila Fuentes-Gualotuña, Jenny Ruales

**Affiliations:** 1 Programa de Doctorado en Ciencia y Tecnología de Alimentos, Departamento de Ciencia de Alimentos y Biotecnología, Facultad de Ingeniería Química y Agroindustria, Escuela Politécnica Nacional, Quito 170525, Ecuador; roberto.ordonez@epn.edu.ec (R.O.-A.); paul.vargas@epn.edu.ec (P.V.-J.); 2 Facultad de Salud y Bienestar, Escuela de Nutrición y Dietética, Universidad Iberoamericana del Ecuador (UNIB.E), Quito 170143, Ecuador; 3 Escuela de Gastronomía, Universidad de Las Américas (UDLA), Quito 170513, Ecuador; nicolas.rodriguez@udla.edu.ec; 4 Programa de Maestría en Desarrollo e Innovación en Alimentos, Universidad de Las Américas (UDLA), Quito 170125, Ecuador; 5 Grupo de Investigación Bio-Quimioinformática, Carrera de Ingeniería Agroindustrial, Facultad de Ingeniería y Ciencias Aplicadas, Universidad de Las Américas (UDLA), Quito 170125, Ecuador; 6 Departamento de Ciencias Nucleares, Facultad de Ingeniería Química y Agroindustria, Escuela Politécnica Nacional, Quito 170525, Ecuador; 7 Unidad de Laboratorio y Análisis, Instituto Nacional de Patrimonio Cultural (INPC), Quito 170143, Ecuador; martha.romero@patrimoniocultural.gob.ec; 8 Facultad de Ciencias Químicas, Carrera de Química de Alimentos, Universidad Central del Ecuador, Quito 170129, Ecuador; ravallejo@uce.edu.ec (R.V.-H.); cafuentesg@uce.edu.ec (C.F.-G.); 9 Departamento de Ciencia de Alimentos y Biotecnología, Facultad de Ingeniería Química y Agroindustria, Escuela Politécnica Nacional (EPN), Quito 170143, Ecuador; jenny.ruales@epn.edu.ec

**Keywords:** food, archeology, biomolecules, macronutrients, culture, chemistry

## Abstract

Within the realm of archaeology, the analysis of biomolecules assumes significant importance in elucidating historical dietary patterns and their implications for contemporary contexts. To achieve this, knowledge and tools of both chemistry and archaeology are essential to yield objective outcomes and conduct analyses of archaeological materials for the detection of biomolecules. Usually, only minuscule remnants of ceramic fragments are retrieved from excavations, which limits the feasibility of comprehensive laboratory analysis. This study aimed to establish a protocol for analyzing fatty acids and starch from archaeological food utensils with minimal sample quantities. Various experiments were conducted to replicate preparations that might have occurred in archaeological vessels, aiming to establish the optimal protocol. The analyses were performed using clay griddles, subjecting vegetable oil to varying temperatures for fatty acid assessment. For starch analysis, a series of experiments encompassed diverse forms of potato preparations (pulp, *chuño*, tortilla, carbonization, and freeze-drying) and maize (flour, tortilla, and carbonization). The verification of the experiments was confirmed by conducting identical analyses, as developed in the current study, on authentic archaeological fragments. The principal outcomes of this investigation include the successful extraction of both types of biomolecules using only 0.25 g of the sample, obtained through direct scraping from the vessel. Soxhlet extraction was identified as the most efficient strategy to recover fatty acids. Additionally, a comprehensive protocol for the identification of starch extraction was developed. This study has, for the first time, elucidated two detailed methodologies for the extraction of fatty acids and starch in scenarios in which researchers can obtain limited quantities of archaeological food utensil fragments.

## 1. Introduction

The subsistence of individuals and the pursuit of nourishment at different epochs have extended beyond a mere physiological necessity, operating as a dynamic force steering daily existence for humanity. This process has not only cultivated social interactions among diverse communities, but has also served as a foundational pillar fostering the development of cultural identity, both during one’s lifetime and in the contemplation of mortality [1]. In the field of archaeological inquiry, there is an interest in the identification of biomolecules associated with food in ancient artifacts, accomplished through chemical analysis. This approach facilitates a more objective comprehension of past dietary habits. Nowadays, collaboration between archaeology and other disciplines is essential, broadening the scope of analytical tools to analyze various compounds (macronutrients, micronutrients, and natural toxins, among others) and understanding factors that affected ancient populations. By integrating different disciplines, it becomes feasible to enhance our understanding of the relationship and significance of archaeological discoveries within the context of the history, societal evolution, and cultural practices of ancient civilizations [2].

Over time, macromolecules have been the subject of archaeological research to establish correlations between traditional diets, nutritional habits, and cultural contexts [3,4]. Numerous studies have associated the Paleolithic era with elevated protein consumption, which is a notion that is still referenced when discussing this historical period [5,6]. However, dietary diversity was probably more extensive than previously hypothesized, since humans likely required a significant intake of carbohydrates. It has even been suggested that Neanderthal populations depended significantly on diets abundant in carbohydrates to satisfy their daily energy requirements [7,8].

Among the dietary biomolecules, carbohydrates and lipids seem to hold particular significance. Several archaeological studies have focused on analyzing the presence of starch and fatty acids in utensils traditionally utilized for food preparation, grinding, or storage [9]. Starch derived from various food sources tends to accumulate within surface irregularities on vessel walls, and this allows the material to maintain properties like a crystalline structure, and birefringence over time [10]. In addition, ceramics can absorb triglycerides (the main component of fat and vegetable oils), thus facilitating their preservation over extended durations [11,12]. Factors that could contribute to the preservation of biomolecules encompass the presence of conditions such as reduced water activity (which inhibits the enzymatic activity responsible for the degradation of starch and lipid compounds), close-to-neutral pH, and relatively lower ambient temperatures. Under these conditions, macronutrients could potentially be identified within archaeological artifacts, even in utensils that have been cleaned by archaeologists. On the other hand, if these parameters show instability, starch and lipids may undergo hydrolysis or oxidation, resulting in the degradation and non-preservation of macromolecules over time [13,14].

The identification of carbohydrates and lipids provides a means to partially discern the potential dietary habits of past civilizations. For instance, the Andean region of South America is renowned for its pronounced starch-rich diet. Within this context, the main constituents of the diet have been ascribed to maize (*Zea mays*) and potato (*Solanum* spp.). Maize domestication was initiated in highland Mexico approximately around 10,000 to 9000 cal BP [15], originating from the Teocintle, and subsequently disseminated to both North and South America. The earliest archaeological findings in South America are concentrated in Peru [16,17], Ecuador [18,19,20,21], and Colombia [22,23], with origins dating back to approximately 9870 to 8600 cal BP. The origin of the potato lies within the American continent. The earliest archaeological evidence of wild potato use dates to between 10,900 and 10,100 BC in Utah (North America) [24,25,26] and the domesticated potato dates to 7800 BC found in the Casma valley (Peru) [27]. Genomic data indicate a potato domestication start date of around 10,000 BC, with a single domestication origin in the southern Peruvian Andes [28,29,30]. One of the significant traditional potato-based preparations in the Andean region is *chuño* (freeze-dried potato). This age-old preservation technique involves positioning the *chuño* at elevations exceeding 3800 m above sea level, where nighttime temperatures descend below 3 °C. Thawing occurs during daylight hours via sublimation. This procedure yields a product referred to as *chuño negro*. Additionally, there exists a variation of the traditional method known as *chuño blanco*, Tunta, or Moraya [31,32,33].

Continuing the discussion on the significance of identifying macronutrients to investigate the dietary patterns of ancient civilizations, research conducted in the Andean region suggests the consumption of various foods rich in fatty acids. For instance, in the present-day territory of Quito, Ecuador, archaeological evidence indicates the consumption of foods notable for their lipid content. There were many examples of potential fatty-acid-rich diets including fish, duck, rabbit, deer, guinea pig, llama, mouse, skunk, opossum, dog, snail, quinoa, and amaranth [34].

In ancient civilizations, food served as a foundational element, making the identification of dietary characteristics essential for comprehending these societies. In the context of the Andean region, the identification of fatty acids (FAs) and starch offers an objective means to discern the dietary habits of its inhabitants. Numerous studies have focused on the chromatographic analysis of fatty acids. However, the main limitations of this technique are (a) the low content of preserved organic matter (less than 1 g of sample), due to the high susceptibility of lipids (including FAs) to pre- and post-depositional chemical changes [35,36], (b) the high cost, and (c) the destruction of irreplaceable archaeological material. For the identification of starches, a specialized protocol has been employed to facilitate observation through optical microscopy. Nevertheless, some studies use substantial sample sizes, ranging from 1 to 5 g (g), which may not be suitable because of the scarce amount of this type of sample and also from an economical perspective.

This study aimed to conduct diverse firing experiments on a clay griddle to recover fatty acid and starch molecules, using a limited sample size. Various methodologies employed in diverse archaeological investigations were examined to identify the optimal procedure for recovering fatty acids and starch. Subsequently, following the identification of the optimal methodology, its effectiveness was verified by applying the extraction of these molecules to authentic archaeological samples.

## 2. Materials and Methods

In this study, an experimental design was devised to incorporate fatty acids and starch into a clay griddle, to subsequently determine the optimal methodology for their identification. For the fatty acid analysis, various plates and fragments underwent firing with vegetable oil. For starch analysis, two staple foods that have been integral to the Andean diet for millennia were selected: potato and maize. To identify potato starch, the raw material underwent analysis, and various preparations were conducted, including pulp, black *chuño*, white *chuño* flour, white *chuño* tortilla, charred white *chuño* tortilla, scrapings from clay griddles post-cooking, and freeze-dried potato. For maize, the selections consisted of maize flour, two varying types of tortillas, carbonized maize tortilla, and scrapings from the plates. To confirm the efficacy of the methodology, four samples of archaeological vessels were utilized to corroborate the experiments concerning the analysis of fatty acids and starch.

### 2.1. Raw Materials

In the context of the fatty acid experiment, a refined oil from a local brand, which included a blend of soybean oil and palm olein, was acquired from a supermarket. Potato (*Solanum tuberosum*) of the “*Super Chola*” variety, with a genetic origin of ([Rosita × Curipamba) × *S. phureja*]) × normal Chola) × Chola 1,2,3), was utilized. For maize (*Zea mays*), the improved INIAP-124 Mischa variety was obtained. These samples were purchased from a market situated in the city of Quito. The white *chuño* flour was acquired from a Peruvian market and subsequently transported securely to Ecuador in an airtight container. All samples collected were aseptically transported in sterile bags to both the laboratory and analysis department of the National Heritage Institute of Ecuador (INPC) and the food research and development laboratory of the School of Gastronomy at the Universidad de las Américas (UDLA). The potatoes and maize were subjected to a thorough rinsing using distilled water, followed by drying at a temperature of 50 °C in an oven. Clay griddles were procured from a market in Quito, intended for the replication of firing processes observed in pre-Hispanic artifacts. Before the start of the experiment, these slabs underwent a series of preparatory steps. Specifically, they were subjected to heating and subsequent rinsing with distilled water, disinfection utilizing ethanol, and final drying within an oven at 65 °C. This procedure was employed to eliminate any potential traces of external starch presence [37].

### 2.2. Sample Processing

#### 2.2.1. Vegetable Oil

Two clay griddles were employed for the experiment, with one of the plates intentionally fractured into small pieces to replicate the fragments commonly unearthed in archaeological settings. These fragments were then placed inside the intact clay griddle and filled with oil. Following this, the griddle was introduced into an oven set at a constant internal temperature of 120 °C for 30 min. Subsequently, a vacuum process was initiated, with the procedure repeated three times for intervals of 30 s each, maintained at a pressure of 0.1 MPa. The fragments containing the impregnated oil were then placed into a sterile bag.

#### 2.2.2. Potato-Based Preparations

To obtain various potato preparations, a sterilized scalpel was employed to peel and extract the pulp. (1) Pulp: Potato pulp was utilized for the analysis; this procedure was executed immediately preceding the analysis to prevent enzymatic browning. (2) *Chuño*: to replicate the traditional method of preparing black *chuño*, the procedure outlined by de Haan et al. (2010) and Melton et al. (2020) [32,38] was followed, with adaptations made to suit laboratory conditions. The process involved freezing the potatoes in a refrigerator at −15 °C for 12 h, followed by placement in a dehydrator at 55 °C for 6 h. Subsequently, the potatoes were manually pressed with disinfected hands to remove excess moisture, a step repeated twice. Finally, after the last repetition, the potatoes underwent an additional dehydration phase of 8 h at the same temperature. (3) White *chuño* flour and tortilla with *chuño* flour: The *chuño* flour was utilized directly for analysis, while the culinary preparation involved mixing the flour with water and cooking on the clay griddle. Specifically, the preparation entailed combining the flour with 80% of its weight in water (several experiments were conducted to determine the optimal proportion for achieving a consistent tortilla texture, with stability attained using this water-to-flour ratio). (4) Charred tortilla: A portion of the tortilla was cooked on the clay griddle until it achieved charring. (5) Clay griddle: The plate used for cooking served to collect the starch absorbed during the culinary processes. The surface was cleansed using a sterile brush, and a solution of acetic acid (5%) was applied to eliminate any residual starch present on the surface. (6) Freeze-dried potato: The process entailed initial freezing at −32 °C, succeeded by the application of a vacuum at 0.85 mBar, and subsequent dehydration at 50 °C for 8 h. Following each preparation, the samples were carefully stored in sterile bags for transfer to the laboratory.

#### 2.2.3. Maize-Based Preparations

To prepare the maize samples, the grains were first passed through a mill that had been washed, disinfected, and oven-dried. (1) Maize flour: The resulting maize flour was utilized for both starch analysis and culinary preparations. (2) Maize tortilla: Two baking processes were conducted on a clay griddle, during which various water-to-flour ratios were tested to ascertain the optimal texture for tortilla formation. Water proportions of 80% and 100% relative to the weight of the flour were explored to achieve an optimal texture. (3) Carbonized tortilla: A portion of the tortilla was subjected to cooking until carbonization occurred. (4) Clay griddle: Following the diverse cooking processes, the clay griddle underwent the identical cleaning procedure as in the potato experiment. Subsequently, the samples were stored in sterile bags for subsequent transfer and analysis.

**Figure 1:**
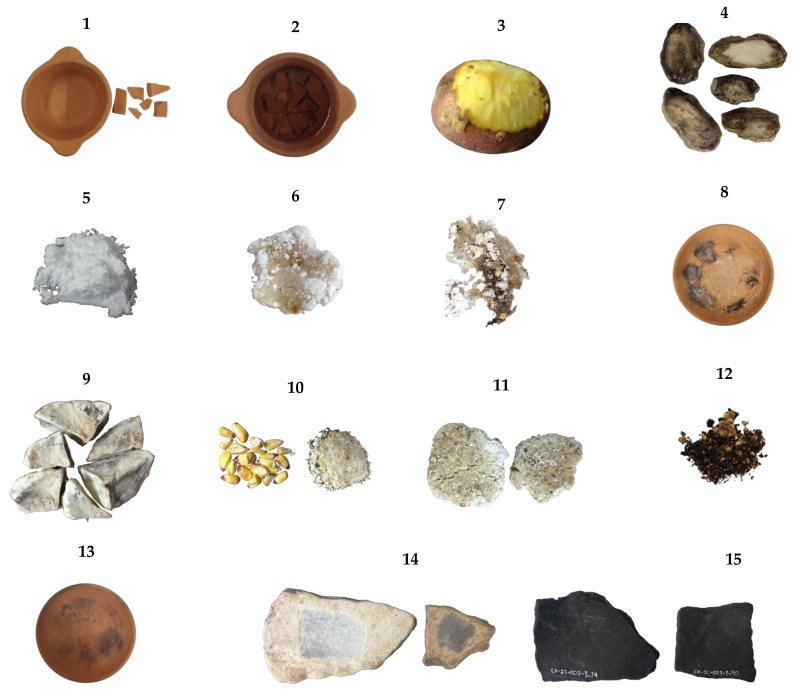
Samples used to analyze starch and fatty acids. 1. Clay griddle and fragments for fatty acid experiment. 2. Clay griddle and fragments with vegetable oil for high-temperature application. 3. Potato pulp. 4. Black *chuño*. 5. White *chuño* flour. 6. White *chuño* tortilla. 7. Charred white *chuño* tortilla. 8. Clay griddle utilized in the process of cooking *chuño* tortillas and their subsequent removal through scraping. 9. Freeze-dried potato. 10. Maize and maize flour. 11. Maize tortillas prepared with flour-to-water ratios of 80% and 100%. 12. Charred maize tortilla. 13. Clay griddle utilized in the process of cooking maize tortillas and their subsequent removal through scraping. 14. Archaeological samples for starch analysis. 15. Archaeological samples for fatty acid analysis.

Figure 1 illustrates the samples employed in the experiment.

### 2.3. Fatty Acids Experiment

Before initiating the current investigation, the research team conducted a series of experiments aimed at analyzing heavy metals in archaeological samples in the UDLA chemistry laboratory. These trials yielded conclusive evidence indicating that favorable outcomes could be achieved using a minimal sample size of 0.25 g. Given this premise, and recognizing that the analysis of heavy metals necessitates highly sensitive analytical approaches where sample quantity is crucial for obtaining accurate outcomes, a sample size of 0.25 g was selected for use to investigate the feasibility of identifying fatty acids and starch.

#### 2.3.1. Sample Collection and Extraction

Following a review of the primary methods utilized in archaeological research, four experiments were undertaken, each employing distinct extraction methodologies. The principal aim was to ascertain the most effective method when dealing with limited sample quantities. The experimental procedures were categorized as follows: (1) Direct scraping of the sample with subsequent Soxhlet extraction; (2) Crushing of the sample followed by Soxhlet extraction; (3) Direct scraping of the sample coupled with Bligh and Dyer extraction; (4) Crushing of the sample along with Bligh and Dyer extraction. Additionally, an analysis of pure oil was conducted, and a control sample was included.

To acquire the sample, fragments of the clay griddle were scraped until the necessary quantity was obtained. All reagents were procured from the laboratory’s designated chemical supplier; these reagents are known for their easy accessibility and wide availability, facilitating the experimental procedures. In experiments 1 and 2, the fatty acid extraction protocol outlined in ISO 1443:1973 (Meat and meat products—Determination of total fat content) [39] was employed. The sample was combined with 2.5 milliliters (mL) of hydrochloric acid (4 N), following which the test tube was heated to its boiling point and maintained at that temperature for one hour. Subsequently, 7.5 mL of distilled water was introduced. The resultant mixture was then filtered through filter paper and the paper was subsequently dried in an oven at 103 °C for one hour. The dried filter paper was then inserted into the thimble of the Soxhlet apparatus, with *n*-hexane (C_6_H_14_) added in a ratio of 1:1/2 relative to the capacity of the extraction tube. The flask of the Soxhlet equipment was heated for 4 h in a water bath (Isotemp–Fisher Scientific—Waltham, MA, USA), after which the solvent in the flask was distilled off.

For samples (3) and (4), the method of Bligh and Dyer, 1959 [40] was used, with minor adjustments for use with the 0.25 g sample size. In this procedure, the sample was mixed with 1.5 mL of distilled water, and then 1.25 mL of a chloroform/methanol solution (2:1 *v*/*v*) was added as a solvent. The mixture was placed in an ultrasonic bath (Cole Parmer Antylia Scientific company—08895-40—Vernon Hills, IL, USA) for 15 min, and this step was repeated twice. The mixture was then centrifuged (Eppendorf (miniSpin)) at 2000 rpm for 5 min. The supernatant was filtered off, and 0.8 mL of distilled water was added and shaken. The organic phase, which was expected to contain the lipid extract, was recovered using a rotary evaporator (Buchi R-300). In addition, a nitrogen stream can be used, as reported in several studies [41].

#### 2.3.2. Derivatization Chromatography

The sample in a flask was treated with a sodium–methoxide solution (CH_3_ONa-0.5 N). A reflux condenser was then attached, and the mixture was heated to boiling for 15 min. Subsequently, chloromethane (CH_3_Cl) was added and maintained at boiling temperature for 10 min. After removal from heat to cool, distilled water was added to aid in transferring the contents to a separatory funnel containing 2 mL of C_6_H_14_. The mixture was stirred and allowed to stand until phase separation occurred. The non-polar phase was subjected to another extraction, followed by a second extraction of the polar phase with an additional 2 mL of C_6_H_14_. The two extractions were combined, and the resulting mixture was dried over 0.5 g of sodium sulfate (Na_2_SO_4_) before filtration. Following this, the samples underwent analysis utilizing a gas chromatograph equipped with a flame ionization detector (FID) (model YL Instrument 6500 GC). The fatty acid methyl esters (FAMEs) were compared against the reference standard FAME MIX C4-C24 (Sigma Aldrich, Oakville, ON, Canada). The percentage of each fatty acid recovered was compared with the fatty acids found in the pure oil (RCPO). To assess the recovered fatty acids, the open-source software R (4.3.3 version) was utilized.

#### 2.3.3. Verification of the Methodology

Once the optimal method for fatty acid analysis was established, an experiment verification was conducted using available fragments of archaeological vessels. Two archaeological samples (AS) were used; the procedure involved direct scraping of the samples and subsequent Soxhlet extraction, following the aforementioned steps. Fatty acid analysis was carried out on two fragments from an archaeological vessel associated with food storage, following the identified funerary context. These fragments were discovered during excavations in the Rumipamba region of Quito and were radiocarbon-dated to the Formative period of the pre-Hispanic Quito chronology, which spanned from 1500 to 500 BC [34]. The Metropolitan Institute of Heritage in Quito supplied two samples in hermetically sealed containers for fatty acid analysis. The fragments were subsequently transported to the laboratory at the INPC, where, within a sterile environment, each piece was scraped using a fresh scalpel for each sample. A fine powder, possibly containing residues of food biomolecules, was obtained. For each fatty acid analysis, 0.25 g of this powder was utilized. The procedure that exhibited the most favorable outcomes for the identification of fatty acids in this study was subsequently employed for the analysis of the samples.

### 2.4. Starch Experiment

#### 2.4.1. Sample Collection

To acquire samples from clay grids and archaeological fragments, an initial cleaning process using a sterile brush was conducted. Subsequently, for the extraction of potential biomolecules contained within the vessels, the following methods can be employed: direct scraping of the vessel or vessel fragment [42], or immersion of the vessel fragment in distilled water followed by ultrasonic treatment [43]. For this study, the direct scraping method was chosen due to its ability to provide the entire sample without dilution. Additionally, a control sample was analyzed by scraping a clay griddle that had not undergone any firing process.

#### 2.4.2. Proposed Methodology

Upon reviewing the literature detailing the procedure for starch extraction from archaeological artifacts, it is evident that many studies draw from the protocol outlined by Atchison and Fullagar (1998) [44]. However, it should be noted that these studies often deviate from the protocol’s exact steps and incorporate various modifications. In this context, we conducted a review of 8 distinct studies [20,25,43,45,46,47,48,49]. Following this, a series of experiments was conducted, altering various parameters, to establish an extraction protocol based on the most favorable result.

The following section presents the protocol utilized for the identification of starch in archaeological samples: (1) Sterilize the designated area using a 5% acetic acid solution. (2) Place 0.25 g of the archaeological sample into a 2 mL sterile plastic tube. (3) Overlay the sample with 1.25 mL of cesium chloride (CsCl) solution prepared to possess a specific gravity within the range of 1.79–1.80 g/cm^3^ (it is advisable to utilize a density-measuring instrument as the exact quantity of CsCl required to achieve this density might not be intuitively calculable). (4) Agitate for 10 min. Subsequently, perform centrifugation at 3000 rpm for 20 min (during this stage, particles with densities below that of CsCl will rise to the surface. Starch exhibits an average specific density of 1.5 g/cm^3^). (5) Transfer the buoyant fraction to a fresh 2 mL sterile plastic tube. (6) Step 6 is discretionary: in the case of seeking a purer sample for microscopic examination, the addition of CsCl to 1.5 mL is possible, followed by the repetition of steps 4 and 5. (7) Include distilled water until a total of 1.5 mL is reached (this action is taken to lower the specific gravity of the CsCl). (8) Centrifuge at a speed of 9000 rpm for a duration of 8 min. (9) Pour off 0.5 mL of the upper floating fraction (given the adjusted density, starch, being lighter, precipitates in this mixture). (10) Add distilled water to reach a total volume of 1.5 mL and centrifuge at 5000 rpm for 5 min. (11) Remove 1 mL of the floating fraction by decanting. (12) Perform a repetition of step 10. (13) Dispose of 1.25 mL of the floating fraction. (14) The residual material is deposited onto a slide along with a drop of glycerol. (15) Observe under a microscope.

#### 2.4.3. Microscopy

An Olympus BX53F optical microscope equipped with an integrated Infinity 2 digital camera possessing polarized capabilities was employed. Each plate underwent initial comprehensive scanning using a 10× objective. Upon starch identification, a 40× objective was employed, and the images showcased in the results section were captured. Prior to each measurement, the area was sterilized using a 5% acetic acid solution, and the microscope lenses were cleansed with alcohol. Ultimately, the starch images acquired were compared with the reference collection to facilitate a comprehensive description.

#### 2.4.4. Verification of the Methodology

Similar to the verification process for fatty acid analysis, the proposed methodology for starch analysis was also verified. Two archaeological vessel fragments, supplied by IMP, were selected for this verification. These vessels, associated with potential food preparation, were discovered within a funerary context during the excavation of the Llano Chico site in Quito, Ecuador. Radiocarbon dating conducted on these artifacts situates them within the Regional Development period of the pre-Hispanic Quito chronology, dating from 500 BC to 500 AD [32]. To procure the samples for analysis, the fragments were scraped using the same method as employed in the fatty acid experiment. A 0.25 g sample was extracted, and the protocol outlined in this research was followed for starch extraction and identification.

## 3. Results

### 3.1. Fatty Acids

**Figure 2:**
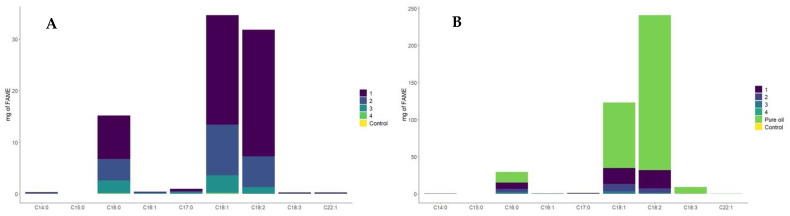
Amount of fatty acid methyl esters retrieved from the 4 experiments and pure oil. mg of FAME: milligrams of fatty acid methyl esters. Main fatty acids retrieved in the experiment: Palmitic acid: C16:0; oleic acid: C18:1; linoleic acid: C18:2. (**A**): Comparison of fatty acids retrieved in the 4 experiments. (**B**): Comparison of fatty acids from the 4 experiments and pure oil.

The milligrams (mg) of fatty acid methyl esters were acquired from 2 mL of *n*-hexane in each experiment. The principal fatty acids constituting the oil encompassed palmitic, oleic, and linoleic acids. One of the objectives of this study was to compare the recovery of fatty acids using various methods with a sample size of 0.25 g. Therefore, our focus was not on describing each fatty acid; instead, the primary outcome was to analyze the recovery rates of the major fatty acids present in the samples. To facilitate the evaluation of the results, Table 1 and Figure 2 exclusively showcase the predominant fatty acids that were identified. Numerous minor fatty acids, due to their tiny presence and potential to interfere with the analysis, have been omitted. Table 1 presents the primary fatty acids found within the oil. Based on the collected data, it can be determined that experiment 1 resulted in the highest percentage of recovery.

Notably, all of the methodologies exhibited recovery for each fatty acid, except for experiment 4. When analyzing fatty acids present in diminutive archaeological fragments, it is noteworthy that experiments 1, 2, and 3 are all suitable options. Each of these methodologies is capable of detecting the presence of these molecules within the sample. Upon obtaining the experimental results, the extraction of fatty acids from archaeological samples was executed through the utilization of Procedure 1 methodology, employing 0.25 g of the sample, and, subsequently, the retrieval of various fatty acids was achieved (Table 1).

In Figure 2A illustrates that experiment 1 produced the most favorable outcomes, followed by experiment 2, whereas experiment 4 showed a lower recovery percentage. Additionally, it is noteworthy that the control sample showed no values in the analysis, and experiment 3 showcased values falling between sample 2 and 4. Within Figure 2B, the inclusion of pure oil enables the differentiation from the experimental outcomes. A unified figure encapsulating all data was omitted because direct oil measurements resulted in elevated quantities of fatty acid methyl esters.

### 3.2. Starch

For starch analysis, the protocols outlined in the starch comparative material guide by Pagán-Jiménez (2015) [10] and other relevant research were followed. In both experiments, the control sample did not produce detectable results.

#### 3.2.1. Potato

**Figure 3:**
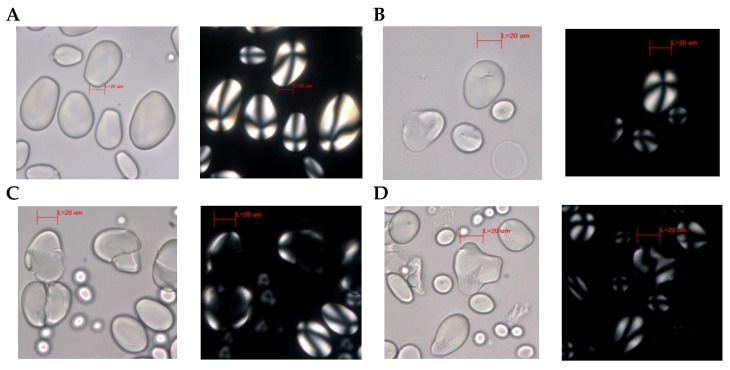

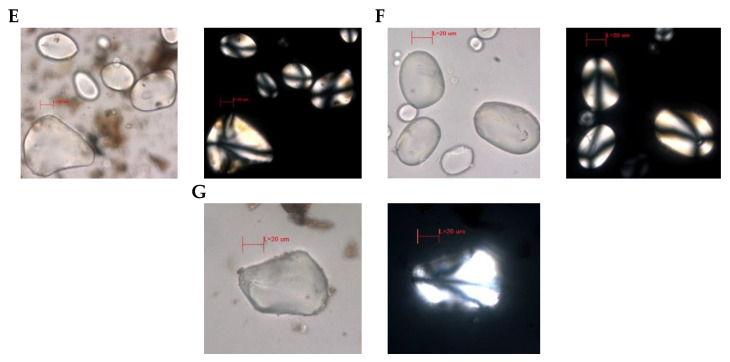
Starch retrieved through diverse experimental procedures applied to potato, observed using both bright-field view and polarized light microscopy. Gray background corresponds to the visual representation of potato starches observed under bright-field view, while the black background reflects their appearance under polarized light. The starch samples in the experiments are as follows: (**A**) potato pulp, (**B**) black *chuño*, (**C**) white *chuño* flour, (**D**) *chuño* tortilla with 80% water incorporation by weight, (**E**) carbonized tortilla, (**F**) freeze-dried potato, and (**G**) scraping from a clay griddle. Red line represents 20 µm measurement.

Figure 3 illustrates the outcomes acquired for potato starch in various preparations, with the successful recovery of starch observed in all conducted experiments. Figure 3A shows an untreated potato sample, exhibiting distinctive characteristics of the species. The granule is simple and elliptical, exceeding 20 µm in size. It features a non-articulated surface with a wavy margin, a closed off-center hilum, and the absence of cracks or external edge lines. This granule lacks the common internal-to-external laminate pattern observed in this species. Under polarized light, the extinction cross displays pronounced curved arms, as documented in previous studies [50,51,52].

Figure 3B corresponds to black *chuño*, showcasing granules resembling those found in the native potato. However, a granule displaying distinct deformations is evident, potentially arising from prolonged drying [53], thereby making the assessment of its extinction cross unfeasible. Furthermore, an A-shaped linear crack is discernible at the hilum. Figure 3C,D provide an overview of the outcomes on *chuño* flour and the resulting tortilla preparation. These share common typical traits associated with starch subjected to these procedures. Notably, partially discernible lamellae, morphological distortions attributed to both drying and the gelatinization process during tortilla formulation, are observable. Furthermore, select granules exhibit diminished birefringence upon polarized light, along with modified extinction cross shapes [32,54]. In Figure 3E, a display of multiple starch granules exhibits structural transformations to a polymorphic state. This examination pertains to the analysis of the fully carbonized tortilla. Notably, circular fissures are also discernible in the hilum region, while certain instances reveal changes in the extinction cross, characterized by the emergence of undulating arms, pressure facets, and altered patterns of extinction cross arms [32]. Figure 3F illustrates the freeze-dried potato, presenting a flattened profile with the absence of discernible lamellae. Linear cracks are noticeable in the hilum region, while the extinction cross exhibits conspicuously curved arms, akin to the characteristics observed in Figure 3A. Figure 3G portrays a substantial ellipsoidal form with an eccentric hilum, characteristic of potato remnants typically found after years of underground storage [51]. Morphological alterations are apparent, indicative of various preparation and cooking procedures that the granule has undergone. Additionally, slight concentric rings are observed in a regular pattern. Under polarized light, diminished birefringence is evident, accompanied by the presence of distorted wavy arms [54].

#### 3.2.2. Maize

**Figure 4:**
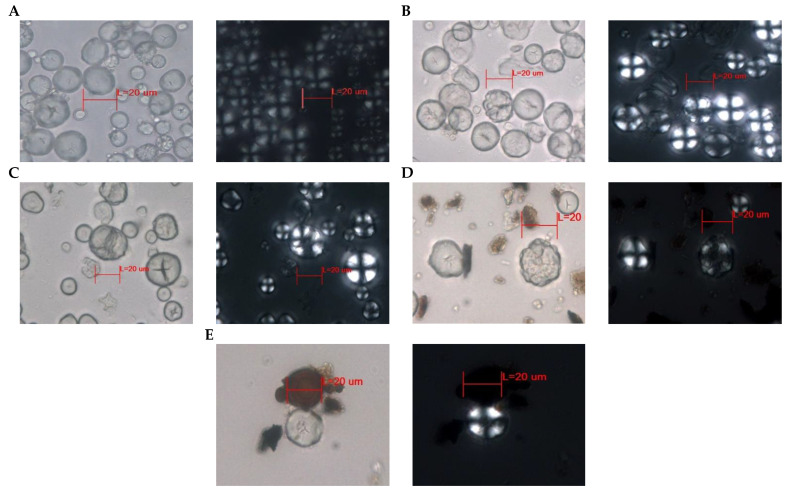
Starch retrieved through diverse experimental procedures applied to maize, observed using both bright-field view and polarized light microscopy. Gray background corresponds to the visual representation of maize starches observed under bright-field view, while the black background reflects their appearance under polarized light. The starch samples in the experiments are as follows: (**A**) ground maize, (**B**) maize tortilla with 80% water incorporation by weight, (**C**) maize tortilla with 100% water incorporation by weight, (**D**) charred maize tortilla, and (**E**) scraping from a clay griddle. Red line represents 20 µm measurement.

Similar to potato starch, maize starch was found in all preparations. Figure 4A illustrates maize flour that underwent grinding without thermal processing. The starch’s general characteristics are evident: the average size measures 20 µm, and the granules possess a straightforward structure, manifesting circular or oval shapes with occasional deformations. The hilum is generally obscured, although it can be discerned in certain granules through Y, T, X, and transverse line fissures, whether centrally or eccentrically positioned. The formation of any laminate is not distinguishable; instead, the granules exhibit simple margins and bear distinct imprints of multiple pressure facets and double borders. When viewed under polarized light, the distinct extinction cross associated with maize starch is observed, varying between centric and eccentric variants, featuring well-defined straight arms [45].

Figure 4B,C, present the outcomes of maize starch cooked with 80 and 100% water incorporation. Here, we observe noticeable morphological distortions in the granules along with a distinct double edge. The fissures and cavities that emerge in maize starch due to thermal treatments are evident; specifically, this analysis has led to the development of prominent linear A-shaped, star-shaped, and cross-shaped fissures. Additionally, pressure facets are observable, coexisting with both simple and compound structures. When examined under polarized light, the full spectrum of transformations undergone by the granules becomes apparent. A mixture of centric and eccentric extinction crosses is observed, primarily characterized by straight arms, albeit wavy arms can also be detected. The loss of birefringence is noticeable in certain granules that display deformations in the two images. Notably, in Figure 4C, additional arms can be observed in the extinction cross of one of the granules exhibiting the most substantial fissure [45,55]. In Figure 4D, the alterations in starch structure resulting from the carbonization process are depicted. The starch granules exhibit varying degrees of distortion, ranging from complete disfigurement to partial deformation characterized by the emergence of striations. Additionally, a distinctive T-shaped crack is observed. Despite the evident diminishment of birefringence, both straight and curved extinction crosses remain discernible, manifesting in both centric and eccentric variations. In Figure 4E, we find starch recovered from the scraping of the clay griddle; small fissures can be seen, and pressure facet and edges have been marked in some parts of the granule. The extinction cross is in the centric position with straight arms despite the loss of birefringence in some areas of the granule [56].

#### 3.2.3. Starch in Archaeological Fragments

**Figure 5:**
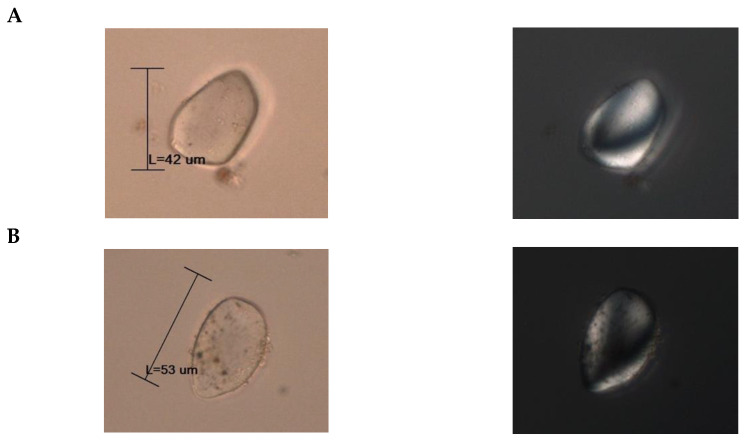
Starch recovered from archaeological vessel fragments. Possible achira: *Canna indica* (syn. edulis) starch. (**A**): Starch identified in fragment 1. (**B**): Starch identified in fragment 2 from the Quito Regional Period samples. Bright-field images are displayed on the right side. Images under polarized light microscopy are shown on the left side.

In the examined fragments of archaeological pottery, the analysis revealed the presence of two distinct starch varieties, as depicted in Figure 5. Both starches exhibit similar properties when observed under polarized light microscopy, suggesting a common dietary source. Figure 5A,B, when viewed without polarized light, display comparable particle size; however, discernible disparities in their structural characteristics become evident. These variations may be attributed to thermal processing, soaking, or mechanical crushing, as previously suggested.

Figure 5A displays a starch particle with a subtly triangular morphology, whereas Figure 5B exhibits an elliptical–triangular configuration and displays indications of degradation, potentially attributed to thermal influence. In both starches, neither the hilum nor the laminate structure is discernible, and there are no apparent fractures; furthermore, they exhibit undulating margins. The extinction cross-sections for both starch types show a shared feature, characterized by an eccentric, cross-shaped pattern with undulating extensions in the distal region.

Upon a thorough examination and comparison of the starch samples retrieved in this study with the modern comparative material outlined in Pagán-Jiménez’s (2015) *Guide to Ecuadorian Comparative Materials* [10], we can conclude that these starches bear a resemblance to the achira starch *Canna indica* (syn. edulis). Another characteristic is that these starches are indicative of cultivated rather than wild variants of the plant. This observation suggests a high level of agricultural expertise among the people during the Regional Development period in Quito-Ecuador.

## 4. Discussion

The quality control of samples during experiments holds significant importance. While in our study, the contamination of certain types of starch or fatty acids may not be relevant since their identification would reveal discrepancies, in archaeological samples, such contamination could potentially lead to misinterpretation errors. Among the studies that have investigated the presence of fatty acids in archaeological contexts, various protocols have been employed to successfully identify these compounds. An integral focus of this study was to ascertain that effective outcomes can be achieved through the direct scraping of the Soxhlet extraction method of sample collection. This approach holds significance as it sheds light on a potentially underutilized procedure, thereby offering a valuable alternative for future investigations in which such a methodology may not have been previously considered. This recommendation can be substantiated upon the successful verification of the method using authentic archaeological specimens. Transferring a contemporary experimental protocol to materials dating back hundreds of years can pose inherent challenges. Nevertheless, we have demonstrated the feasibility of this endeavor within the scope of this study. Despite being present in relatively diminished quantities in comparison to the experimental samples, these fatty acids were successfully identified, even in the context of samples dating back thousands of years.

As previously discussed, numerous investigations have been conducted on the analysis of fatty acids in archaeological vessels. Upon reviewing these studies, it becomes evident that the majority of them utilize intact vessels or large fragments to procure samples [9,41,57,58,59,60]. However, there exists no consensus on a standardized protocol for identifying fatty acids, as different methods are employed in each study. This discrepancy poses a challenge, particularly when only small fragments are available for analysis, as procedures requiring a large sample volume may not be feasible.

The laboratory, where the analysis of fatty acids was conducted, has extensively dealt with vegetable oil analysis. Specifically, palmitic, oleic, and linoleic fatty acids consistently emerge as predominant components in these analyses. This observation is in alignment with other research findings, substantiating the presence of these specific fatty acids within these oils [61,62]. The recovered percentages of fatty acids from the archaeological samples affirm the method’s efficacy. This enables comparisons with other investigations where this macronutrient has been detected in archaeological contexts. For instance, previous studies in the Andean region have identified palmitic, stearic, capric, and undecanoic acids in vessel samples [41,60,63], which aligns with the fatty acids found in our investigation’s samples. Such findings suggest a potential correlation between the outcomes of our study and those of vessels from Quito. In archaeological contexts, quantification may not always be imperative. Given the temporal context of buried vessels, the mere identification of present fatty acids suffices for discerning the potential dietary components.

An illustrative instance is the investigation by Pecci et al. (2016) [64], wherein they executed a controlled firing experiment involving glazed vessels and concurrently examined the fatty acid compositions in archaeological pottery. In their experimental firing scenario, they incorporated substances like wine, olive oil, and lentisk oil. Correspondingly, akin to the present study, a single repetition was undertaken for each experiment. Notably, for lipid extraction and recovery, they adopted the same methodology employed in this current research for experiments 3 and 4. Their adept application of this approach enabled the identification of principal fatty acids within both the oils and the archaeological samples. Despite the author’s success in detecting fatty acids within their analyses, there exists the potential for heightened recovery rates through the utilization of our investigation’s protocols (1) or (2). Even with a reduced sample size, such as the one employed in our study (0.25 g), this enhancement could have been achieved. It is worth noting that their original analysis employed a sample size of 1 g.

Establishing the feasibility of conducting the analysis with a sample size of 0.25 g holds significant implications for future research, rendering various fragment sizes viable for investigation. Additionally, our study elucidates that the analysis of fatty acids can be accomplished solely through sample scraping, obviating the necessity for distilled water or a solvent to extract sediment from the vessel. This observation gains further importance considering that, as indicated by our results, such additional steps could potentially impact the overall recovery of fatty acids.

In the study conducted by Craig et al. (2011) [65], a sample size of 1 g was employed, and the extraction procedure followed our suggested protocols (3) and (4). Their investigation encompassed the analysis of 220 potsherds. It is conceivable that had the researchers adopted our proposed method, more favorable outcomes for their analysis could have been achieved. Additionally, an alternative approach has been identified that eliminates the need for fragment crushing.

Research by Casanova et al. (2022) and García-Granero et al. (2022) [9,66] opted to crush entire portions of potsherds and utilized sample quantities ranging from 2 to 10 g. However, the viability of replicating such studies is constrained by practical concerns stemming from the potential fragmentation of vessels, as highlighted earlier. Research employing methodologies similar to those outlined in experiments 1, 2, and 3 of our study (though with minor deviations in certain extraction procedures) have also been carried out by other researchers [60,67,68]. However, it is noteworthy that none of these investigations delved into the utilization of the minimal sample size demonstrated in our study. These studies employed sample quantities ranging from 4 to 10 g, or, in some cases, did not specify the gram measurements employed.

In the study conducted by García et al. (2017) [69], an analysis was undertaken on the bottoms and rims of amphorae, primarily utilized for the transportation of inadequately filtered or unfiltered olive oil within the Guadalquivir valley during the II Iron Age. Within this context, a significant accumulation of fatty matter residues was discovered on the rims of these vessels. For their analytical procedure, the sample underwent a crushing process; however, specific details concerning the sediment removal process were not provided (although a drill might have been employed). Similar to our study, a sample quantity of 0.25 g was utilized for analysis, and the sampling and extraction methodology closely resembled our experiment 2. In cases where the samples did not originate from fatty matter, a discernibly reduced quantity of fatty acids was retrieved compared to samples with higher fatty matter content. For forthcoming investigations involving amphorae where a substantial accumulation of fatty matter residues is absent, it could be beneficial to consider the utilization of our experiment 1. This approach is likely to yield a greater recovery of mg of fatty acid methyl esters, thereby enhancing the analytical outcomes.

Finally, we can contrast our experimental approach with a reference source discussing methods for the analysis of fatty acids in archaeological materials: “Analytical strategies for discriminating archaeological fatty substances from animal origin” by Regert (2011) [70]. In this reference, it is suggested that sufficient lipids can be extracted from 2 g of material sourced from ceramic fragments measuring approximately 4 cm^2^. However, it is important to note that obtaining this quantity of sample from such a fragment size might entail a substantial degree of fragmentation. Our investigation demonstrates the viability of achieving meaningful results using a significantly reduced quantity of grams, thereby negating the necessity of extensively altering or potentially compromising the integrity of the sample, particularly in cases of smaller-sized fragments. The author also specifies the extraction procedure akin to our experiments 3 and 4, while neglecting any mention of the potential utility of employing the Soxhlet method, which our study has revealed to yield superior outcomes. Experiment 4 exhibited unremarkable percentages of fatty acid recovery despite utilizing the same sample as the other experiments. One plausible explanation for this outcome could be that the method requires a larger sample size to yield significant results. Consequently, it may not be advisable to employ this method when only a limited sample is available.

In the realm of starch analysis, a comprehensive protocol was formulated by examining several studies. Our proposed methodology is designed to accommodate starch analysis using a mere 0.25 g of sample. This approach encompassed various experimental conditions involving maize and potato, as well as the direct scraping of vessels used for cooking starchy foods. Regarding the procedural aspect, we have developed a detailed protocol to ensure its replicability in any investigative context. Similar to the verification process undertaken for the fatty acid experiment, we conducted a verification using authentic archaeological vessel fragments. This verification underscores the protocol’s adaptability for use across diverse contexts.

Detecting remnants of starch from various food sources in archaeological artifacts provides insight into the significance of diet in ancient times. Understanding the dietary patterns of past civilizations enables us to comprehend how each community sourced food and cultivated new crops as part of their daily sustenance. Furthermore, it sheds light on the societal value attached to these food items. For instance, studies have revealed the presence of cocoa starch in certain archaeological excavations. This discovery has contributed to our comprehension of the significance of cocoa in the pre-Hispanic era. The cultivation of cocoa and the consumption of chocolate beverages were not merely culinary practices, but were also intertwined with rituals and notions of abundance, as cocoa was revered as a divine food [49,71].

While certain studies provide scant details regarding starch extraction methodologies [56,72], or fail to explicitly outline their extraction procedures [73], there are investigations such as those conducted by Jaime-Pagán that showcase the experimentation steps. Within Jaime-Pagán’s publications pertaining to archaeological starch analysis, an illustrative example can be found in the “Protocol for the Double Extraction of Starch Grains and Phytoliths from Sediments” authored by Santiago-Marrero and Pagán-Jiménez (2023) [74]. This protocol outlines the methodology for analyzing archaeological starches, tailored for a sample size of 4 g. It suggests adjusting the quantities if a smaller sediment sample is under examination. However, in light of our current study’s findings, we consider that for this, an experiment like the one developed in the present investigation should be carried out, in order to determine whether it is feasible to use a smaller amount of sample. This recommendation stems from the fact that the protocol in question employs reagents such as sodium hexametaphosphate from the outset, and it remains uncertain whether these can effectively facilitate extraction with a smaller sample volume. Likewise, within the outlined protocol, the authors do not expound upon the methodology employed for procuring the sediment they utilize. In contrast, our investigation confidently asserts that direct scraping yields favorable outcomes. Furthermore, there is no mention of the preference for hand washing and disinfection over the use of sterile gloves, despite a prior recommendation by one of the authors in a previous study on starch extraction protocols [45].

We have developed a protocol with the objective of defining each step and ensuring its reproducibility. It is important to note that certain protocols, like the one proposed by Kovárník and Beneš (2018) [75], may offer a wealth of information; however, important small details may be missing that would not allow replicating in a direct way in a study.

Subsequent to the establishment of the protocol, an examination was conducted to ascertain the alterations that potato and maize starch may undergo when subjected to diverse treatment modalities. This analysis revealed characteristic traits, encompassing morphological deformities leading to the formation of striations, various forms of fissures, and diminished birefringence under polarized light. These findings parallel the typical outcomes observed in archaeological starch extractions, as evidenced in previous studies such as Cagnato et al. (2022); Kovárník and Beneš (2018) and Mickleburgh and Pagán-Jiménez (2012) [75,76,77]. These alterations predominantly arise from the process of gelatinization, which starch undergoes upon interacting with water at varying temperatures. Consequently, potato starch experiences its prime period of morphological transformation within the temperature range of 61 to 75 °C, while corn starch encounters this phase between 64 and 77 °C. The extent of granular changes hinges on multiple factors that instigate starch modification, including parameters like contact duration, temperature, composition of food mixtures, and the specific cooking methodology employed [78].

An alternative culinary technique that imparts alterations to starch morphology is drying. In our investigation, this approach was employed on potatoes to produce *chuño* (both black and white variants). This involves subjecting the starch to extended drying periods, resulting in distinct structural impairments within the extracted starch due to the process [53]. However, such changes are absent in freeze-dried potatoes, as their preservation process, involving sublimation at lower temperatures, safeguards the integrity of tissue, texture, and visual aspects of the food [79].

Within our carbonization process, we discerned the occurrence of granular distortions, a result attributed to the combined effects of gelatinization and elevated temperatures. These factors can induce a misshapen expansion in numerous granules [32]. Additionally, it is imperative to recognize fully cooked starch, as archaeological settings often yield charred roots, tubers, or roasted foods that might have undergone significant alterations, leading to the loss of several characteristic features [73]. The carbonized starches extracted from potato and maize in our study exhibit a correlation with those identified in ceramic and lithic artifacts, as documented by Pagán-Jiménez et al. (2016) and Zimmermann (2021) [20,80].

Among the ancient starch grains obtained from the analysis of archaeological artifacts on the island of Trinidad and central French Guiana in the research of Pagán-Jiménez et al. (2015) [47], they indicate that their image G Figure 9 is an unidentified tuber starch of considerable size that could not be identified. This image shares similarities with Figure 3 (G) of our study, which would allow us to identify that potatoes, sweet potatoes, or directly *chuño* were consumed there, a product of a possible commercial exchange of the time.

It is noteworthy that starch was successfully extracted through scraping clay griddle used for cooking. In cases of repeated cooking within the same utensil, there exists the potential for an accumulation of starch granules over time [13]. This aspect holds significance, considering ancient practices of using the same cooking implements daily. The utilization of our protocol, requiring minimal sample quantities, offers the possibility of identifying such ancient culinary usage patterns.

Limitations and challenges may arise when employing the proposed methodologies on archaeological samples. One such challenge is the potential for unexpected results due to the absence of biomolecules in the selected sample, highlighting the possibility that macronutrients may not be present. If a larger quantity of sample is available, the analysis can be repeated multiple times or adjusted based on the sample amount. In starch analysis, to ensure that no starch has been lost, the liquid discarded during the process from step 9 can be examined under a microscope. Additionally, external contamination is another concern that could occur, so strict adherence to cleanliness protocols is imperative to mitigate such issues. For instance, in executing the starch protocol, it is advisable to avoid using nitrile gloves. In a study conducted by Ciofalo et al. (2018) [45], a significantly altered starch was detected in a glove, despite its designation as sterile and powder-free. This implies the potential for cross-contamination. Therefore, it is recommended to maintain short fingernails and adhere to thorough hand-washing and disinfection procedures to mitigate any risks. While the experiments conducted have provided insights into potential optimal protocols for identifying fatty acids and starch, it is important to note that these findings could vary depending on factors such as the material of the utensils, the historical period, geographical location, and other parameters influencing the preservation capacity of biomolecules within the samples.

Finally, this study offers a comprehensive protocol for the identification of fatty acids and starch. Each method is designed for identifying biomolecules in archaeological artifacts. While previous studies have detected fatty acids and starch, this research provides a detailed overview encompassing all facets to enable replication of the method in any laboratory. Studies referencing extraction and identification methodologies often omit important aspects or fail to provide detailed descriptions of each step.

## 5. Conclusions

The outcomes derived from this study underscore the viability of detecting fatty acids and starch within archaeological remnants, even when working with minute sample fractions. This investigation reveals that satisfactory outcomes can be achieved with a minimum of 0.25 g of the sample material.

The results derived from this research represent an advancement in the examination of biomolecules within the archaeological domain. This study has provided insight into the dispensability of substantial sample quantities for the successful extraction of fatty acids and starch.

This study can be replicated through an exploration of additional sediment extraction methods, including the utilization of distilled water combined with an ultrasonic bath. By undertaking such an approach, it becomes possible to ascertain and compare the extent of biomolecule extraction achieved.

This study provides a perspective for future research to integrate archaeology and food science. In contemporary research, interdisciplinary collaboration is essential. Integrating various scientific disciplines enables us to obtain objective findings, thus enhancing our understanding of the history and culture of our ancestors.

## Figures and Tables

**Table 1:** Milligrams of major fatty acid methyl esters and percentage recovery compared to pure oil from each experiment and fatty acids retrieved from archeological samples.

Fatty Acid		Pure Oil	1 (mg)	% RCPO	2 (mg)	% RCPO	3 (mg)	% RCPO	4 (mg)	% RCPO	Control	AS1 (mg)	AS2 (mg)
Capric	C10:0	-	-	-	-	-	-	-	-	-	-	0.017	0.018
Undecanoic	C11:0	-	-	-	-	-	-	-	-	-	-	0.020	0.017
Lauric	C12:0	-	-	-	-	-	-	-	-	-	-	0.004	-
Myristic	C14:0	0.26	0.18	71%	0.09	35%	0.05	21%	0.00	1%	0.00	0.007	0.010
Pentadecanoic	C15:0	0.04	0.02	53%	0.01	26%	0.00	13%	0.00	0%	0.00	0.014	0.010
Pentadecenoic acid (cis-10)	C15:1	-	-	-	-	-	-	-	-	-	-	0.109	0.093
Palmitic	C16:0	14.25	8.41	59%	4.22	30%	2.40	17%	0.16	1%	0.00	0.026	-
Palmitoleic	C16:1	0.26	0.09	35%	0.18	67%	0.13	49%	0.01	2%	0.00	0.003	0.003
Heptadecanoic	C17:0	0.00	0.47	0%	0.21	0%	0.27	0%	0.03	0%	0.00	-	-
Heptadecenoic acid (cis-10)	C17:1	-	-	-	-	-	-	-	-	-	-	0.007	-
Stearic	C18:0	-	-	-	-	-	-	-	-	-	-	0.038	0.028
Oleic	C18:1	88.25	21.23	24%	9.79	11%	3.40	4%	0.21	0%	0.00	-	-
Linoleic	C18:2	208.94	24.53	12%	5.93	3%	1.33	1%	0.01	0%	0.00	-	-
Gamma- Linolenic	C18:3	8.81	0.12	1%	0.07	1%	0.04	0%	0.01	0%	0.00	-	-
Erucic	C22:1	0.27	0.14	50%	0.07	25%	0.04	16%	0.00	0%	0.00	-	-

1: Experiment 1 (direct scraping of the sample with Soxhlet extraction). 2: Experiment 2 (sample crushing with Soxhlet extraction). 3: Experiment 3 (direct scraping with Bligh and Dyer extraction). 4: Experiment 4 (sample crushing with Bligh and Dyer extraction). mg: milligrams. % RCPO: percentage recovery compared to pure oil. AS: archaeological samples. Fatty acids with negligible concentration were not considered.

## Data Availability

All data generated by this research have been included in the article. For any assistance, it is possible to contact the corresponding authors.
